# Intravaginal Probiotics in Transition Dairy Cows: A Randomized Multi-Farm Field Trial on Health and Milk Production

**DOI:** 10.3390/vetsci13060595

**Published:** 2026-06-18

**Authors:** Eduardo Rosales Barahona, Andre Luiz Garcia Dias, Ashley Egyedy, Burim N. Ametaj

**Affiliations:** Department of Agricultural, Food and Nutritional Science, University of Alberta, Edmonton, AB T6G 2P5, Canada; ebarahon@ualberta.ca (E.R.B.); garciadi@ualberta.ca (A.L.G.D.); egyedy@ualberta.ca (A.E.)

**Keywords:** lactic acid bacteria, *Lactobacillus sakei*, *Pediococcus acidilactici*, endometritis, vaginal microbiome, periparturient dairy cow, antimicrobial alternative, milk yield

## Abstract

Uterine infections (metritis and endometritis) are a leading cause of culling and reproductive failure in transition dairy cows, and antibiotic-resistant Gram-negative pathogens limit conventional therapy. This randomized, controlled, multi-farm field trial evaluated whether four intravaginal infusions of a host-adapted lactic acid bacteria (LAB) cocktail (*Lactobacillus sakei* FUA3089, *Pediococcus acidilactici* FUA3138, *P. acidilactici* FUA3140; 10^8^–10^9^ cfu/dose) at −3, −2, +3, and +4 weeks relative to calving reduce periparturient disease and improve milk production. A total of 526 pregnant cows (426 Holstein, 100 Jersey) from four commercial Alberta farms (automatic-milking, parlor, and certified-organic systems) were block-randomized within farm and parity to TRT1 (saline; *n* = 175), TRT2 (saline + skim milk; *n* = 176), or TRT3 (LAB cocktail in saline + skim milk; *n* = 175). Uterine infection incidence was assessed by Metricheck™ mucus scoring and transrectal ultrasonography at +3 and +4 weeks postpartum. Across the principal peripartum infectious outcomes, TRT3 showed a consistent protective effect: uterine infection incidence was lowest in TRT3 (18.8% vs. 25.1% in pooled controls; OR = 0.69; 95% CI, 0.44–1.09; an approximately 25% relative reduction; exact *p* = 0.12), and this metritis signal was additionally supported by a repeated-measures mixed model accounting for farm, parity, and week (*p* = 0.0175), although the Bonferroni-adjusted pairwise contrasts were tendencies (adjusted *p* ≈ 0.12), and the effect did not differ by parity (treatment × lactation interaction, *p* = 0.97). Subclinical mastitis was numerically lower in TRT3 than in pooled controls (5.3% vs. 8.9%; OR = 0.57; 95% CI, 0.27–1.24; exact *p* = 0.16), whereas retained placenta, milk fever, displaced abomasum, and lameness showed no clear cow-level treatment effect in the cow-level exact analyses. Milk yield increased significantly in multiparous cows, which produced 4.6 L/day more milk than TRT1 and 3.22 L/day more than TRT2 over the first 50 days in milk (*p* < 0.01 for both contrasts; treatment × parity interaction, *p* = 0.01). No effect was seen on milk composition, uterine involution, or reproductive performance. The trial supports intravaginal LAB as a candidate antibiotic-free prophylactic whose response depends on farm- and cow-level contexts and whose mechanisms require confirmation through microbiological and metabolic measurements.

## Simple Summary

After calving, dairy cows are highly vulnerable to uterine infections, mastitis, and milk fever, disorders that compromise welfare, reduce milk yield, and often require antibiotic treatment. Because antibiotics raise resistance concerns and are largely prohibited on organic farms, safe alternatives are increasingly needed. We tested whether infusing beneficial bacteria (probiotics) into the vagina twice before and twice after calving could help prevent these problems. The trial involved 526 Holstein and Jersey cows across four commercial Alberta dairy farms, including a certified organic herd. Cows receiving the probiotic had fewer uterine infections, and older (multiparous) cows produced more milk. These findings suggest that intravaginal probiotics are a promising, antibiotic-free way to support the health and productivity of dairy cows, although larger studies are needed to confirm these benefits.

## 1. Introduction

The transition period, broadly defined as the three weeks before and the three weeks after parturition, is the most biologically and economically demanding phase of the dairy cow’s productive cycle and a period of profound vulnerability to infectious disease. Clinical metritis affects 15–25% of dairy cows in North American herds, while subclinical endometritis is estimated to affect 30–40% of cows that appear clinically normal [1,2,3]. In Alberta, Canada, reproductive failure attributable directly or indirectly to uterine infections accounts for 61% of culling decisions in commercial dairy herds [4]. The economic burden per case of clinical metritis has been estimated at USD 240–884 (mean ≈ USD 511) [5], underscoring the need for effective preventive interventions. Recent work continues to identify the periparturient inflammatory and metabolic environment as a major driver of postpartum disease in modern dairy systems.

The pathogenesis of postpartum uterine disease is intimately linked to the immunosuppressive milieu of the periparturient period. Parturition disrupts mucosal barrier integrity, allowing ascending contamination of the uterine lumen by bacteria from the caudal reproductive tract and perineum [6,7]. Concurrent peripartum immunosuppression, mediated by elevated glucocorticoids, reduced lymphocyte proliferation, impaired neutrophil oxidative burst activity, and suppression of pattern-recognition receptor signaling, substantially impairs the host’s capacity to resolve this contamination [8]. Principal uterine pathogens, including *Escherichia coli*, *Trueperella pyogenes*, *Fusobacterium necrophorum*, and *Prevotella* species, exploit this immune deficit through biofilm formation, endotoxin-mediated dysregulation of prostaglandin F2α, and direct endometrial invasion [3,6,9,10]. Gram-negative lipopolysaccharide (LPS) released from uterine pathogens translocates across compromised mucosal barriers into the systemic circulation, where it triggers hepatic acute-phase protein synthesis and contributes to the cluster of periparturient metabolic diseases, including mastitis, milk fever, displaced abomasum, and ketosis, that share a common inflammatory and endotoxemic substrate [11,12,13].

Current therapeutic approaches to postpartum uterine disease remain inadequate. Systemic antibiotic therapy, predominantly ceftiofur or oxytetracycline, achieves microbiological cure in only 40–60% of clinical metritis cases, carries withdrawal periods for milk and meat, and contributes to the global crisis of antimicrobial resistance [14,15,16]. Intrauterine preparations, including iodine, chlorhexidine, and prostaglandin analogs, have shown inconsistent efficacy and risk disruption of the regenerating endometrial epithelium [14,16]. In certified organic dairy systems, where antibiotic administration is restricted to cases of verified therapeutic necessity and is limited to two courses per animal per year [17], these limitations are especially acute and leave producers with virtually no evidence-based prophylactic option for uterine disease prevention. The development of safe, effective, antibiotic-free interventions adoptable across both conventional and organic production systems is therefore a recognized priority.

Lactic acid bacteria isolated from the reproductive tracts of healthy dairy cows are a biologically rational candidate intervention, although the bovine vaginal microbiome differs from that of humans: the vaginal pH of healthy postpartum cows is near neutral (mean 7.3 ± 0.63) [18], rather than the acidic environment that favors *Lactobacillus* dominance in the human vagina, and lactobacilli are present at low abundance in the bovine tract [19]. The therapeutic rationale for intravaginal LAB in cattle is therefore best understood as an exogenous competitive intervention rather than a restoration of a dominant endogenous community. The host-adapted strains used here, *L. sakei* FUA3089 and *P. acidilactici* FUA3138/3140, were originally isolated from the vaginal microbiota of healthy multiparous dairy cows and characterized as producers of pediocin AcH/PA-1 [19]. When administered intravaginally, such strains transiently colonize the vaginal mucosa and exert antimicrobial activity through two complementary routes: direct chemical action via lactic acid and hydrogen peroxide secretion [20,21] and ecological displacement of ascending uterine pathogens through niche occupancy and adhesion inhibition [22,23,24]. In vitro evidence further indicates that *Lactobacillus* and *Pediococcus* strains modulate NF-κB-dependent pro-inflammatory cytokine release, inhibit *E. coli* adhesion to bovine endometrial epithelial cells, and attenuate prostaglandin E2 production by endometrial stromal cells under pathogen challenge [25,26,27], suggesting a dual antimicrobial and immunomodulatory mode of action.

Prior work from our laboratory demonstrated that intravaginal infusion of this LAB cocktail decreased uterine infection incidence by approximately 45% in single-herd Holstein trials using six-dose regimens [28,29]. Independent groups have subsequently reported comparable benefits with two-dose prepartum protocols, with effect sizes varying across herds and management systems [30,31]. However, these studies were conducted on single farms under uniform management conditions, and the six-dose regimen imposes a labor burden that limits practical adoption. Whether a streamlined four-dose protocol could achieve comparable efficacy across multiple herds, breeds, and management systems, including organic systems, has not yet been evaluated. The present study was designed to test the hypothesis that four intravaginal doses of the LAB cocktail administered at −3 and −2 weeks prepartum and +3 and +4 weeks postpartum could decrease the incidence of periparturient diseases, improve reproductive performance, and enhance milk production in transition dairy cows across conventional and organic commercial farms in Alberta, Canada.

## 2. Materials and Methods

### 2.1. Animals and Experimental Design

All experimental procedures were approved by the University of Alberta Animal Care and Use Committee for Livestock (AUP No. 00001811) and conducted in accordance with the Canadian Council on Animal Care guidelines. The study was conducted between June 2014 and December 2016 on four commercial dairy farms in Alberta, Canada. The reporting of this randomized controlled trial follows the principles of the REFLECT (Reporting Guidelines for Randomized Controlled Trials in Livestock and Food Safety) statement [32].

**Figure 1:**
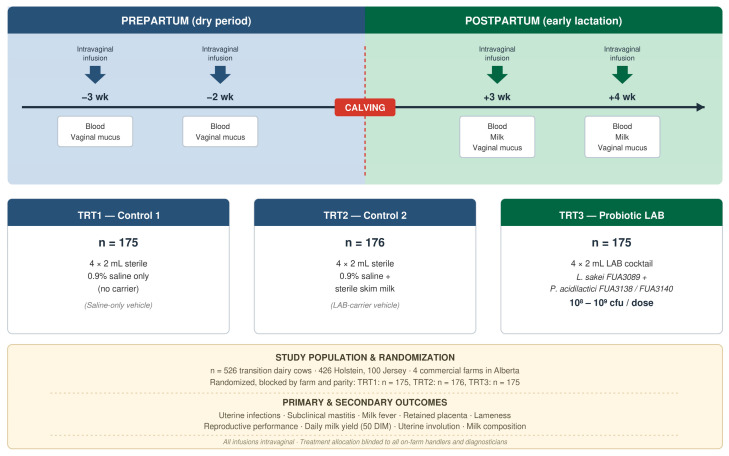
Study design and timeline. Transition dairy cows (*n* = 526) were randomized to three intravaginal treatment groups administered at −3 and −2 weeks prepartum and +3 and +4 weeks postpartum. TRT1: sterile 0.9% saline (control); TRT2: sterile 0.9% saline + sterile skim milk (control); TRT3: lactic acid bacteria (LAB) probiotic cocktail (*Lactobacillus sakei* FUA3089, *Pediococcus acidilactici* FUA3138, *Pediococcus acidilactici* FUA3140; 10^8^–10^9^ cfu/dose). Circles (BM): blood + vaginal mucus sampling; squares (BMM): blood + milk + vaginal mucus sampling; diamonds: intravaginal infusion time points.

A total of 526 pregnant transition dairy cows (426 Holstein and 100 Jersey) from four commercial dairy farms in Alberta were enrolled and block-randomized within farm and parity strata to one of three intravaginal treatment groups: TRT1—sterile 0.9% saline (saline-only vehicle; *n* = 175); TRT2—sterile 0.9% saline + sterile skim milk (LAB-carrier vehicle; *n* = 176); or TRT3—LAB probiotic cocktail (*Lactobacillus sakei* FUA3089, *Pediococcus acidilactici* FUA3138, *P. acidilactici* FUA3140; 10^8^–10^9^ cfu/dose; *n* = 175) reconstituted in the same saline + skim-milk carrier as TRT2. Each cow received four 2 mL intravaginal doses of her assigned treatment, administered at −3 and −2 weeks prepartum and +3 and +4 weeks postpartum (Figure 1). Thus, TRT3 cows received four doses of the LAB cocktail, each containing approximately 10^8^–10^9^ cfu/dose, for a total administered exposure of approximately 4 × 10^8^ to 4 × 10^9^ cfu per cow across the treatment period. The two vehicle arms (TRT1, TRT2) were included to disentangle the effect of LAB from any carrier effect of the skim-milk component: TRT2 contains the skim-milk-plus-saline carrier without LAB, whereas TRT1 contains saline only. The three-arm design therefore tested four intravaginal doses per cow across three treatment conditions. Random assignment was generated by the project supervisor using a computer-generated randomization sequence stratified by farm and parity. Allocation concealment was maintained by holding the randomization key with the project supervisor; on-farm personnel administering the treatments, all clinical evaluators, microbiology staff, and statistical analysts were blinded to treatment allocation until completion of all data collection. The 180-cow subset used for vaginal mucus sampling, uterine involution assessment, and reproductive performance (described in Section 2.4, Section 2.5 and Section 2.6) was a pre-specified per-farm random selection of 45 cows from each farm (15 per treatment arm per farm; 135 Holstein from Farms A, C, and D; 45 Jersey from Farm B), drawn from the parent cohort and equally balanced across treatment arms within each farm. Eligibility required pregnancy confirmed by transrectal ultrasonography, expected calving within the recruitment window, and the absence at enrollment of traumatic injury (including severe leg injuries), udder disorders, enzootic bovine leukosis with enlarged superficial lymph nodes, or other intercurrent disease. Eligible cows excluded for these reasons before the first prepartum dose were replaced by another cow from the same farm and parity stratum so that target enrollment per arm was met. No post-randomization losses to follow-up occurred after the first intravaginal dose: every randomized cow received her assigned four-dose regimen and completed primary outcome assessment at +3 and +4 weeks postpartum, and all 526 cows were included in the primary outcome analysis. Sample size for the primary outcome (uterine infections) was calculated a priori using SAS PROC POWER: with 175–176 cows per arm, the study provided >80% power at α = 0.05 (two-sided) to detect a 10-percentage-point absolute reduction in uterine infection incidence (e.g., from 37% in pooled vehicle controls to 27% in TRT3), an effect size considered clinically meaningful for periparturient disease prevention. Sample size for the 180-cow subset was also determined a priori, prior to cow selection, by power calculation for the principal subset outcomes (uterine involution, Metricheck™ score, and first-service conception rate): at α = 0.05 and 80% power, 15 cows per treatment arm per farm allows detection of a standardized mean difference of Cohen’s d = 1.06 in uterine horn diameter between TRT3 and a single vehicle arm (corresponding to ≈1.6 cm at an assumed within-arm SD of 1.5 cm [3,33]) and a between-arm difference of approximately 25 percentage points in binary outcomes such as Metricheck score ≥ 2 or first-service conception (Cohen’s h ≈ 0.51). When pooled across the four farms, the 60-cows-per-arm subset provides 80% power to detect a smaller effect of d = 0.52 in horn diameter (≈0.8 cm) and a 21–25 percentage-point between-arm difference in binary outcomes; these thresholds are within the range of clinically meaningful effect sizes reported in the prior bovine uterine involution and reproductive performance literature.

The enrollment, randomization, allocation, follow-up, and analysis populations are summarized in the REFLECT-style flow diagram provided as Figure S1 in the Supplementary Materials.

The LAB cocktail and the skim-milk vehicle were prepared as previously described [28,29] by the Department of Agricultural, Food and Nutritional Science laboratory at the University of Alberta. Briefly, both the LAB cocktail (delivered as a freeze-dried preparation in skim-milk powder) and the skim-milk vehicle (TRT2) were reconstituted in sterile 0.9% saline immediately before infusion and stored at 4 °C until use, with use occurring within 24 h of reconstitution. Each dose was delivered as a 2 mL intravaginal infusion using a sterile single-use catheter. Before each infusion, the perineum was cleaned with a 4% chlorhexidine solution and dried with disposable paper towels. The same infusion procedure was used for all three treatment arms by the same trained personnel, who remained blinded to treatment allocation.

### 2.2. Dairy Farms and Their Management Systems

This study was conducted at four free-stall commercial farms (designated A, B, C, and D for confidentiality) located in the province of Alberta, Canada. Dairy farms operate under a supply-managed production system, but farmers implement their own management system to reach the desired production parameters. Farms A, B, and C collect milk under a voluntary milking system (VMS), also known as a robotic milking system, in which milk is collected from cows at any time over a 24 h period. Farm D is a parlor dairy system where milk is collected three times a day at approximately 04:00, 12:00, and 18:00 h by farm staff. All farms offer a total mixed ration (TMR) on an ad libitum feed and water basis. Each farm formulates its TMR according to its own nutritional requirements, following the National Research Council guidelines [34].

Farm A collects milk under an automatic milking system (AMS; DeLaval VMS Voluntary Milking System, Nisku, AB, Canada; DeLaval International, Nisku, AB, Canada), milking around 150 Holstein cows. Farm B is a certified organic dairy farm and collects milk under an AMS (BouMatic, Madison, WI, USA), milking around 150 Jersey cows. Farm C collects milk under an AMS (Lely Industries N.V., Maassluis, The Netherlands), milking around 150 Holstein cows. Farm D uses a parallel parlor milking system (BouMatic, Madison, WI, USA) to collect milk three times per day, milking around 400 Holstein dairy cows.

Because each farm represents a unique combination of breed (Holstein vs. Jersey), milking system (AMS vs. parlor), and certification status (conventional vs. organic), these factors are fully confounded with farm in this multi-site design. Farm-stratified analyses (Section 3.2) describe between-farm patterns descriptively but cannot disentangle the contributions of breed, milking system, and certification status to any observed between-farm differences. This constraint is revisited in the Discussion when interpreting the farm-level findings.

### 2.3. Diagnosis of Clinical Diseases and Clinical Monitoring

All cows were monitored for six periparturient diseases (metritis, endometritis, subclinical mastitis, retained placenta, milk fever, and lameness) and for displaced abomasum by trained on-farm personnel and the attending veterinarian of each farm using standardized case definitions. Disease incidence was recorded prospectively from −3 weeks prepartum through +4 weeks postpartum.

For the purpose of this study, the composite outcome “uterine infections” is operationally defined as the sum of clinical metritis diagnosed within the first 21 days postpartum plus clinical endometritis diagnosed at +3 and +4 weeks postpartum, in accordance with the Sheldon et al. case definitions [3]; pyometra, perimetritis, and parametritis are not included in this composite. Clinical metritis is defined as the presence of an abnormally enlarged uterus on transrectal palpation accompanied by a fetid, watery, red-brown uterine discharge within 21 days postpartum, with or without systemic signs of illness. Clinical endometritis is defined by the presence of purulent or mucopurulent vaginal discharge at +3 or +4 weeks postpartum, scored using the Metricheck™ device (Simcro, Hamilton, New Zealand) on a 0–3-point scale [35], with scores ≥ 2 considered positive. Subclinical mastitis is defined as a quarter-level somatic cell count (SCC) >200,000 cells/mL [36] in the absence of clinical signs (no visible milk abnormality, no quarter swelling, no systemic illness), measured weekly between +1 and +4 weeks postpartum. We note that subclinical mastitis in the present study is defined cytologically by elevated SCC, without confirmatory microbiological identification of causative pathogens; the term “subclinical mastitis” is used throughout in this strictly cytological sense [37,38]. Milk fever (clinical hypocalcemia) is defined by recumbency, cool extremities, decreased mentation, and clinical response to parenteral calcium therapy. Retained placenta is defined as the failure to expel fetal membranes within 24 h postpartum. Displaced abomasum is diagnosed by simultaneous auscultation and percussion (“ping”), confirmed by clinical signs of reduced feed intake and reduced milk yield. Lameness is scored using the 1–5 locomotion scoring system of Sprecher et al. [39], with scores ≥ 3 considered cases.

Body condition scores were measured using the Dairy Cattle Production system (342–450 A, McGill University and Elanco Animal Health, Indianapolis, IN, USA). Scores were evaluated at −3 and −2 weeks before parturition and at +3 and +4 weeks after parturition, on a 5-point scale with 0.25-point intervals.

### 2.4. Uterine Involution

Evaluation of uterine involution was performed by transrectal ultrasonography and manual palpation at +2 and +4 weeks after parturition on 135 multiparous Holstein cows and 45 multiparous Jersey cows. Diameters of the uterine horns were first measured by rectal palpation, using the evaluator’s finger widths. Evaluation of the horns by palpation was done at the base of the uterine horns, where the bifurcation could be palpated, and divided into the dorsal intercornual ligaments and the ventral intercornual ligament. Evaluation of uterine retraction was done by palpation of the ventral intercornual ligament because of its larger size and thickness in both gravid and non-gravid horns [40].

Additionally, a SonoSite^®^ ultrasound (MicroMaxx, SonoSite Inc., Bothell, WA, USA) fitted with a 7.5 MHz probe was used to measure the diameter of the uterine horns to fully characterize involution by both methods. The decrease in size of the bovine uterus after parturition is known to begin during the first 9 days after calving, with uterine horn diameters between 12 and 14 cm in healthy cows. At around +2 weeks after parturition, the uterine horns can measure between 7 and 8 cm, and at +3 and +4 weeks after parturition, diameters range between 2 and 4 cm in healthy cows [33,41].

Proper uterine regression was declared if, by +2 weeks after parturition, both uterine horns measured less than 8 cm and if, by +4 weeks after parturition, the diameter was less than 4 cm. Normally, the uterus has involuted sufficiently by +2 weeks after parturition to allow rectal palpation. Additionally, uterine fluid collected from the 180 cows (45 cows per farm in the study) was evaluated according to Sheldon (2005) [3], as described in the previous section. Healthy cows present scores between 2 and 3 in the first +2 weeks after parturition and then improve to a score of 1 by +4 weeks after parturition. The diagnostic accuracy of rectal palpation for uterine horn-diameter assessment was evaluated using receiver operating characteristic (ROC) curve analysis in MedCalc (version 16.4.3, 64-bit; MedCalc Software, Ostend, Belgium), with the area under the curve (AUC) interpreted according to Swets [42], where AUC > 0.7 indicates an accurate test; the corresponding figure is provided as Figure S2 in the Supplementary Materials.

### 2.5. Reproductive Performance

Reproductive performance was measured for 135 multiparous Holstein cows (45 dairy cows from each of the three Holstein farms) and 45 multiparous Jersey cows, with treatments equally distributed and animals randomly selected for evaluation. Reproductive performance was assessed based on the first-service conception rate and the cumulative conception rate. The conception rate is the percentage of serviced animals that became pregnant. For unbiased data collection, the inseminator, bull, and management were kept blind to treatment allocation. Pregnancy was confirmed by the farm veterinarian. For the purpose of this study, only cows that exhibited natural estrus were evaluated.

### 2.6. Sampling and Laboratory Analyses

Vaginal mucus was collected from 135 multiparous Holstein cows (45 dairy cows from Farms A, C, and D) and 45 multiparous Jersey cows (from Farm B) and transferred into sterile polypropylene test tubes (Fisher Scientific, Toronto, ON, Canada), then stored at −20 °C.

Milk samples were collected once a week from +1 week to +4 weeks after parturition. Daily milk production was measured for the first 50 days in milk (DIM). Samples were collected after three to four streams of milk had been discarded; 3 mL of milk from the four quarters was collected in a vial containing a preservative tablet and stored at 4 °C until analysis. Milk composition was analyzed for fat, crude protein (CP), somatic cell count (SCC), lactose, milk urea nitrogen (MUN), and total solids (TS). Analysis was performed by the Central Milk Testing Laboratory located in Edmonton, Alberta, using mid-infrared spectroscopy (MilkoScan 605; A/S Foss Electric, Hillerød, Denmark).

### 2.7. Statistical Analyses

All statistical analyses were performed using SAS software (version 9.4; SAS Institute Inc., Cary, NC, USA), following the modeling framework described in [43]. A priori sample-size calculation for the primary outcome (uterine infections) was performed using PROC POWER (SAS 9.4) under the assumptions reported in Section 2.1. Three analytical approaches were applied according to the data type of each outcome: logistic regression for binary disease outcomes, linear mixed models for continuous outcomes, and non-parametric tests for ordinal outcomes.

Binary disease outcomes, including metritis, endometritis, the composite uterine-infections outcome, subclinical mastitis, milk fever, retained placenta, displaced abomasum, and lameness, were analyzed using SAS-based models appropriate for categorical disease outcomes. For cow-level summaries, each cow was considered a case when an event was recorded at either +3 or +4 weeks postpartum, and incidence was calculated using treatment-specific animal denominators rather than the distribution of cases among diseased cows. Unadjusted TRT3-versus-pooled-control comparisons are reported for the major disease outcomes as odds ratios with 95% confidence intervals and exact Fisher *p*-values; a 0.5 continuity correction was applied only when a zero cell occurred. Because this was a multi-farm field trial and disease occurrence varied by farm, lactation, and week, a secondary adjusted SAS model including treatment, farm, lactation, and week was also used to evaluate whether the apparent treatment response remained evident after accounting for these major sources of variation. The adjusted model was used to contextualize the multi-farm treatment response and did not replace the cow-level exact estimates reported in Table 1. Treatment effects on metritis/uterine infection were additionally examined within parity strata (primiparous, multiparous) and within individual farms using least-squares-means pairwise contrasts with Bonferroni adjustment for multiple comparisons. Outcomes with low event counts were interpreted descriptively.

Continuous outcomes, including daily milk yield over the first 50 days in milk (DIM), milk composition variables (fat, protein, lactose, SCC, MUN, total solids), body condition score, and uterine horn diameter, were analyzed using linear mixed models in PROC MIXED, with treatment, week, and the treatment × week interaction as fixed effects and farm as a random effect. The previous 305-day milk yield was included as a covariate in the milk-yield model, and parity was tested as an additional fixed effect, with the treatment × parity interaction retained when significant. Normality of residuals was checked using PROC UNIVARIATE before model fitting. The results from continuous-outcome analyses are reported as least-squares means and standard errors of the mean, together with mean differences for the principal treatment contrasts; odds ratios were not computed for continuous outcomes.

Ordinal outcomes, Metricheck™ vaginal mucus scores (0–3), were analyzed as ordinal rather than continuous variables. Frequency distributions were compared across treatment arms using the Kruskal–Wallis test, with Dunn’s test for pairwise post hoc comparisons. Mucus-score data are reported as stacked frequency distributions rather than as means. Covariance structures for mixed-model analyses were selected to minimize Akaike and Bayesian information criteria. The denominator degrees of freedom were estimated by the Kenward–Roger method. *p*-values < 0.05 were considered statistically significant; 0.05 ≤ *p* ≤ 0.10 was treated as a statistical tendency.

Because breed (Holstein vs. Jersey), milking system, and certification status are fully confounded with farm in this multi-site design (Section 2.2), these factors could not be tested as independent fixed effects and were absorbed into the random farm effect. Farm-stratified analyses are reported for the principal outcomes (Section 3.2) to permit between-farm description without making claims about breed or certification status that the design cannot support.

## 3. Results

### 3.1. Effect of Intravaginal Probiotics on the Incidence of Uterine Infection and Other Periparturient Diseases

Overall cow-level clinical observations and incidences of periparturient diseases for the four dairy farms between +3 and +4 weeks postpartum are presented in Table 1. Complete evaluable disease records were available for 508 cows (TRT1 *n* = 169; TRT2 *n* = 169; TRT3 *n* = 170). Across the principal peripartum infectious and inflammatory outcomes, cows treated intravaginally with the LAB probiotic cocktail (TRT3) showed a consistent protective effect relative to the vehicle controls. TRT3 had numerically lower uterine infection incidence than either control (18.8% vs. 25.4% in TRT1 and 24.9% in TRT2; OR vs. pooled controls = 0.69; 95% CI, 0.44–1.09; exact *p* = 0.12), and this association persisted in a cow-level logistic model adjusting for farm and parity (adjusted OR = 0.69; 95% CI, 0.44–1.10; *p* = 0.12). The same protective effect was observed for subclinical mastitis (5.3% in TRT3 vs. 8.9% in pooled controls; OR = 0.57; 95% CI, 0.27–1.24; exact *p* = 0.16) and, relative to the saline-only control, for retained placenta (4.7% in TRT3 vs. 7.7% in TRT1; OR = 0.59; 95% CI, 0.24–1.47; exact *p* = 0.27). Although none of these individual comparisons reached statistical significance, the point estimates were concordant, each indicating roughly 30–45% lower odds in TRT3, and uterine infection showed the strongest and most consistent reduction. This concordant pattern was further supported for metritis/uterine infection by a repeated-measures mixed model (PROC GLIMMIX) that included treatment, farm, parity/lactation, and week, with repeated weekly observations nested within cow, in which treatment was associated with metritis (*p* = 0.0175); body condition score showed a treatment tendency in the same framework (*p* = 0.054). No protective effect was evident for milk fever, lameness, or displaced abomasum, and the very-low-event outcomes (displaced abomasum, *n* = 2 cows) are reported descriptively only.

Parity-stratified uterine-infection outcomes are presented descriptively in Table 2. The numerically lower uterine-infection incidence in TRT3 was driven mainly by multiparous cows (16.9% in TRT3 vs. 23.5% in TRT1 and 25.8% in TRT2), whereas primiparous cows showed smaller differences among treatments.

Parity-stratified subclinical mastitis and milk-fever outcomes are presented in Table 3 and Table 4, respectively. Subclinical mastitis was numerically lower in TRT3, especially in multiparous cows (5.4% in TRT3 vs. 11.8% in TRT1 and 8.3% in TRT2). Milk fever was observed only in multiparous cows and showed no clear treatment effect in the cow-level analysis. There was no significant difference among treatments regarding lameness.

**Figure 2:**
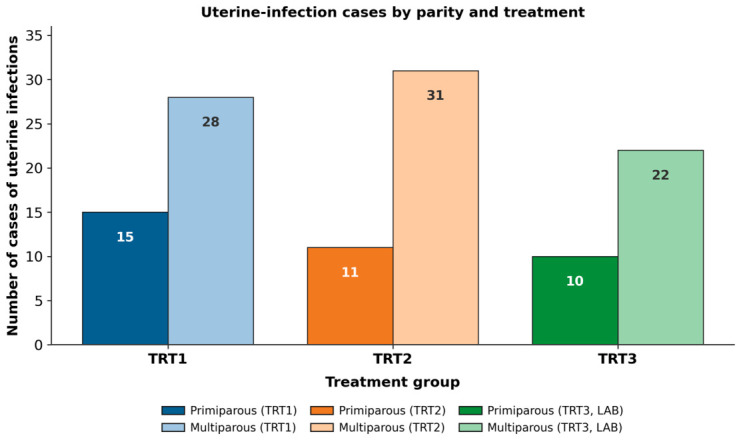
Number of cases of uterine infections after parturition in primiparous and multiparous postpartum dairy cows by treatment group (all four farms combined; *n* = 508 evaluable cows). TRT1: sterile 0.9% saline; TRT2: sterile 0.9% saline + skim milk; TRT3: LAB probiotic cocktail (10^8^–10^9^ cfu/dose). Bars show cow-level case counts; the treatment × lactation interaction was not significant (*p* = 0.97), so no parity-specific significance is indicated.

Odds-ratio analyses for uterine infections by parity are presented in Table 5. These estimates are reported to improve transparency, but the parity-specific analyses should be interpreted cautiously because confidence intervals were wide and some model-based values were sensitive to event counts. Overall, TRT3 showed the lowest numerical incidence among multiparous cows, whereas treatment differences among primiparous cows were small (Figure 2).

Retained-placenta outcomes are presented in Table 6. Cow-level retained-placenta incidence did not differ clearly among treatments (4.7% in TRT3 vs. 7.7% in TRT1 and 4.7% in TRT2; exact *p* = 0.549 for TRT3 vs. pooled controls).

Only two cow-level cases of displaced abomasum were diagnosed across the complete disease-record dataset, so no meaningful treatment effect could be evaluated for this periparturient disease. No significant difference (*p* > 0.05) was found among treatments regarding BCS at any time point.

### 3.2. Treatment Effects Under Different Management Systems

**Figure 3:**
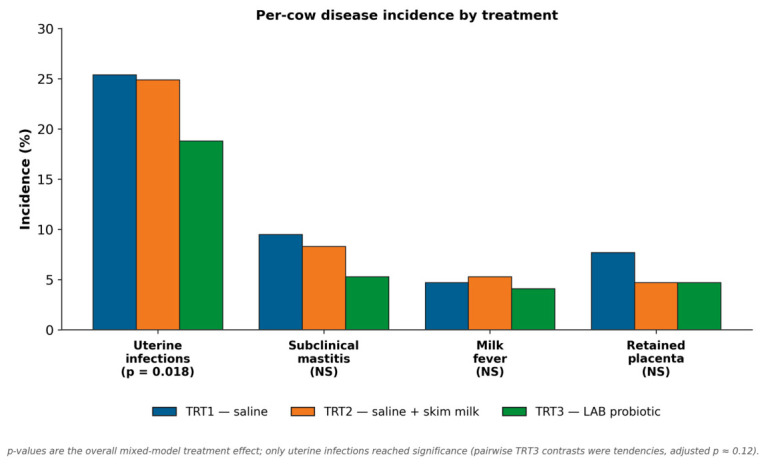
Incidence rates (%) of four periparturient diseases in transition dairy cows treated with intravaginal LAB probiotics (TRT3) or vehicle controls (TRT1, TRT2) across all four participating farms (per-cow incidence; *n* = 508 evaluable cows). Diseases shown: uterine infections (metritis + endometritis), subclinical mastitis (SCC > 200 × 10^3^ cells/mL), milk fever, and retained placenta. In the mixed model, the overall treatment effect was significant for uterine infections (*p* = 0.018); the Bonferroni-adjusted TRT3-versus-control contrasts were tendencies (adjusted *p* ≈ 0.12), and mastitis, milk fever, and retained placenta showed no significant TRT3-specific reduction.

**Figure 4:**
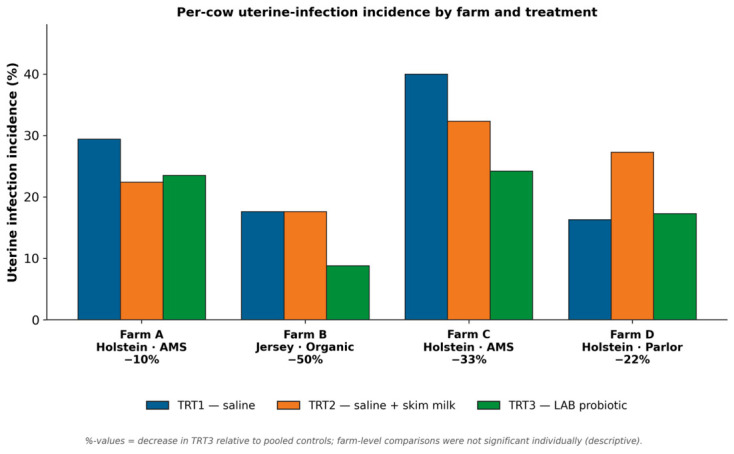
Uterine infection incidence (%) by treatment group across four commercial dairy farms in Alberta. Farm A: Holstein, automatic milking system (AMS); Farm B: Jersey, certified organic, AMS; Farm C: Holstein, AMS; Farm D: Holstein, conventional parlor (3×/day). TRT3 was numerically lowest on three of the four farms; farm-level comparisons did not reach significance individually, given the limited per-herd sample sizes. Percentages indicate the decrease in uterine infection incidence in TRT3 relative to pooled controls within each farm.

The results of clinical observations for disease incidence in cows after parturition for Farms A, B, C, and D are presented in Table 7, Table 8, Table 9, and Table 10, respectively, with the cross-farm comparison summarized in Figure 3. Because farm, breed, milking system, and certification status are confounded in this field design, farm-specific outcomes are presented descriptively and should not be interpreted as independent tests of breed or management system effects. Uterine infection incidence was numerically lower in TRT3 on Farms A, B, and C, whereas Farm D showed similar incidence in TRT1 and TRT3 and higher incidence in TRT2 (Figure 4).

Farm A results are presented in Table 7. On Farm A, TRT3 showed numerically lower cow-level incidence of metritis, subclinical mastitis, retained placenta, and lameness than TRT1. However, exact farm-level comparisons did not reach statistical significance, reflecting the limited number of cows per treatment within a single farm. Because there was only one cow-level case of milk fever and one cow-level case of displaced abomasum on Farm A, those outcomes are presented descriptively.

Farm B results are presented in Table 8. On Farm B, TRT3 showed numerically lower cow-level incidence of metritis and milk fever than pooled controls, but exact farm-level comparisons did not reach statistical significance. Because there were no cases of retained placenta or displaced abomasum, those outcomes are presented descriptively.

Farm C results are presented in Table 9. On Farm C, TRT3 showed numerically lower cow-level incidence of metritis than both vehicle controls. However, exact farm-level comparisons did not reach statistical significance, and the milk-fever and retained-placenta outcomes involved very few cases. Because there were no cases of displaced abomasum, that outcome is presented descriptively.

Farm D results are presented in Table 10. On Farm D, TRT3 had cow-level metritis incidence similar to TRT1 and lower than TRT2. Subclinical mastitis and lameness involved very few cases, and milk fever was numerically higher in TRT3 than in both vehicle controls. These Farm D outcomes emphasize that farm-specific findings should be interpreted descriptively and that confirmatory studies are required before generalizing herd-level effects.

The metritis/uterine-infection signal was the most consistent across analyses. In a repeated-measures mixed model accounting for farm, parity/lactation, and week, with repeated weekly observations nested within cow, treatment was associated with metritis/uterine infection (*p* = 0.0175), with TRT3 showing the lowest model-estimated incidence (18.2%) versus both vehicle controls (≈25.4%); in the Bonferroni-adjusted pairwise contrasts these TRT3-versus-control differences were tendencies rather than significant (adjusted *p* = 0.12 for both TRT1-vs.-TRT3 and TRT2-vs.-TRT3), and the treatment effect did not differ by parity (treatment × lactation interaction, *p* = 0.97). Consistent with this, TRT3 had the lowest metritis incidence on three of the four farms (Table 7, Table 8, Table 9 and Table 10); the per-herd comparisons did not reach significance individually, reflecting the limited per-herd sample sizes, with the most pronounced reduction in the certified-organic Jersey herd (Farm B; 8.8% in TRT3 vs. 17.6% in both controls). Farm and week were strong sources of variation.

### 3.3. Treatment Effects on Uterine Involution

There was no effect of treatment on uterine-horn involution. Overall, the interactions among parity, week, and farm showed no effect on uterine involution. There was no significant difference (*p* > 0.05) among treatments regarding retraction of the right uterine horn (*p* = 0.35) or the left horn (*p* = 0.22) for gravid and non-gravid horns at +2 weeks postpartum. Furthermore, there was no significant effect among treatments regarding the involution of the right uterine horn (*p* = 0.23) or left uterine horn (*p* = 0.23) for gravid and non-gravid horns at +4 weeks postpartum (Figure S2).

**Figure 5:**
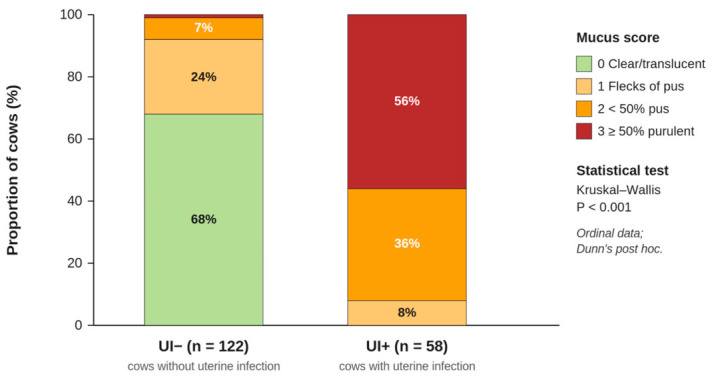
Stacked frequency distribution of Metricheck™ vaginal mucus scores (Sheldon 0–3 ordinal scoring system) by uterine infection (UI) status at +3 to +4 weeks postpartum (*n* = 180 cows; UI− *n* = 122, UI+ *n* = 58). Mucus scores are treated as ordinal data and analyzed using the Kruskal–Wallis test with Dunn’s post hoc procedure. Score 0: clear or translucent mucus; score 1: flecks of pus; score 2: less than 50% pus; score 3: 50% or more purulent material. UI− cows show a strongly right-skewed distribution toward score 0, while UI+ cows are concentrated at scores 2 and 3. *p* < 0.001, confirming a strong positive association between PVD score and uterine infection status, and validating Metricheck™ vaginal mucus scoring as a reliable field diagnostic tool.

The clinical utility of Metricheck™ vaginal mucus (PVD) scoring as a field diagnostic for endometritis was further evaluated in the 180-cow subset (45 cows per farm) and is presented in Figure 5. Cows classified as uterine-infection-negative (UI−; *n* = 122) showed a strongly right-skewed distribution toward score 0 (clear or translucent mucus), whereas UI-positive cows (*n* = 58) were concentrated at scores 2 and 3 (≥50% purulent material), with a highly significant between-group difference (Kruskal–Wallis, *p* < 0.001). This pattern confirms that purulent vaginal discharge scoring is a reliable, non-invasive bedside indicator of postpartum uterine infection under the conditions of the present study.

### 3.4. Treatment Effects on Reproductive Performance

There were no significant effects of treatment (*p* = 0.57) on the first-service conception rate or the cumulative pregnancy rate for Farms A, B, C, and D. The first-service conception rate was 51.0 ± 7.4% for TRT1, 50.0 ± 7.7% for TRT2, and 43.3 ± 8.1% for TRT3. The cumulative pregnancy rate was 84.0 ± 7.7% in TRT1, 86.0 ± 7.9% in TRT2, and 79.0 ± 8.4% in TRT3. No significant TRT × parity interaction was found (*p* = 0.25 for TRT1 and TRT2), although a tendency toward an interaction was noted for TRT3 in multiparous cows (*p* = 0.11).

Odds-ratio analyses for treatments were evaluated for both primiparous and multiparous cows to examine the tendency found in the TRT × parity interaction. No significant effect was found in the odds-ratio test, although cows infused with TRT3 (probiotics) had numerically higher odds of becoming pregnant than TRT1 and TRT2 (OR = 1.16, *p* = 0.79; and OR = 1.61, *p* = 0.38, respectively; Table 11). On average, primiparous cows required 1.5 inseminations per pregnancy compared with multiparous cows, which required 1.9 inseminations (*p* = 0.01).

### 3.5. Milk Production and Composition

Daily milk production was recorded for the first 50 DIM. Milk yield was analyzed using the linear mixed model described in Section 2.7, with treatment, parity, and their interaction included in the model; farm treated as a random effect; and previous 305-day milk yield included as a covariate. There was a significant treatment effect on milk production across Farms A, B, C, and D (*p* < 0.05), and the treatment × parity interaction was significant (*p* = 0.01). Multiparous cows treated with the LAB probiotic cocktail (TRT3) produced 4.6 L/day more milk than TRT1 cows and 3.22 L/day more milk than TRT2 cows over the first 50 DIM (*p* < 0.01 for both contrasts). These values are reported as least-squares mean differences from the linear mixed model; odds ratios are not reported for milk yield because milk yield is a continuous outcome.

The milk composition for all treatment groups is shown in Table 12. Treatment did not have an effect (*p* > 0.05) on milk composition. There were differences in milk components such as protein, lactose, milk urea nitrogen, and total solids at +1 week and +4 weeks postpartum (*p* < 0.05). These components were higher at +1 week than at +4 weeks. There was no significant difference (*p* > 0.05) in the interaction of TRT × parity for any of the components.

## 4. Discussion

The central hypothesis of this study—that a reduced four-dose intravaginal LAB regimen administered at −3, −2, +3, and +4 weeks relative to calving would confer protection against postpartum uterine disease—was supported most strongly for metritis/uterine infection. Across the principal peripartum infectious and inflammatory outcomes (metritis, subclinical mastitis, and retained placenta), TRT3 showed a concordant protective effect, with each point estimate indicating roughly 30–45% lower odds relative to control, even though the individual comparisons did not reach statistical significance after exact pairwise testing. The coherence of this pattern—reinforced for metritis by the adjusted repeated-measures model (*p* = 0.0175)—provides more persuasive support for a true biological effect than any single comparison, whereas the broader non-uterine effects remain exploratory and require confirmation.

The mechanism of protection conferred by the LAB cocktail is likely multifactorial, but the present study did not directly measure vaginal colonization, microbial community composition, qPCR abundance, or pathogen displacement. Therefore, the proposed mechanism should be considered hypothesis-generating rather than demonstrated. Based on prior in vitro and in vivo literature, possible mechanisms include transient niche occupancy, inhibition of pathogen adhesion, local production of antimicrobial metabolites, and modulation of inflammatory signaling [44,45,46,47,48]. However, because no microbiome sequencing, culture-based enumeration, qPCR, or strain-recovery data were generated in the current trial, the present results cannot prove that the observed reduction in uterine disease was mediated by microbiota modulation. Future studies should include longitudinal vaginal and uterine microbiome profiling, strain-specific detection, pathogen culture or qPCR, and assessment of local immune markers to define the causal pathway.

The numerical reduction in subclinical mastitis is an interesting secondary observation; however, after exact pairwise testing and confidence-interval reporting, this outcome should be interpreted cautiously. The overall comparison for subclinical mastitis did not reach statistical significance in the cow-level frequency analysis, and the confidence intervals for pairwise treatment comparisons were wide. A biologically plausible connection between improved uterine health and a lower mammary inflammatory burden may exist through reduced systemic inflammatory signaling, lower environmental contamination, or improved immune competence during early lactation, but the present trial was not designed to establish this pathway. Therefore, although subclinical mastitis contributes to the concordant protective pattern observed across peripartum infectious outcomes, it is best presented as a supportive secondary observation rather than a confirmed independent effect of intravaginal probiotics.

Milk fever is a periparturient imbalance of calcium homeostasis [49,50], and any relationship between intravaginal probiotics and clinical hypocalcemia must therefore be interpreted cautiously. Although inflammation and endotoxin exposure can influence calcium dynamics, feed intake, immune activation, and periparturient metabolic adaptation, the present study did not measure ionized calcium, parathyroid hormone, vitamin D metabolites, endotoxin, or inflammatory mediators at the time of milk-fever diagnosis. In addition, the overall treatment comparison for milk fever was not significant in the cow-level frequency analysis. Therefore, the milk-fever findings should be regarded as a farm-specific, hypothesis-generating signal rather than direct evidence that intravaginal probiotics prevent hypocalcemia. Future studies would need blood calcium measurements and inflammatory biomarkers to test this proposed pathway.

The heterogeneity of treatment responses across farms merits careful interpretation rather than dismissal. TRT3 was numerically lowest for metritis on three of the four farms, with the most pronounced reduction on the organic Jersey herd, although no single farm reached statistical significance. Inter-farm variability in probiotic efficacy is a well-recognized challenge in livestock microbiome interventions and reflects the strong modulatory influence of baseline vaginal microbiome composition [51], housing density, dietary transition protocols, dry-off procedures, and antimicrobial-use history on the capacity of exogenous LAB to establish and maintain competitive dominance [19]. The statistical power constraint at the individual-farm level (*n* ≈ 60–80 per treatment per farm) is a further limiting factor; simulation-based power analyses suggest that farm-level sample sizes of approximately 150 cows per arm would be required to reliably detect a 15–20% decrease in metritis incidence at α = 0.05 and 80% power, underscoring the necessity of multi-site or cluster-randomized designs for definitive efficacy claims in heterogeneous commercial settings [52].

The results from Farm B—a certified organic Jersey herd in which intravaginal probiotics were associated with the largest farm-level reduction in metritis/uterine infection, halving incidence (8.8% in TRT3 vs. 17.6% in both controls)—are of particular interest because organic certification prohibits routine prophylactic antibiotic use and tightly restricts therapeutic antibiotic treatment [17,53], leaving organic producers with minimal evidence-based tools for uterine disease prevention. It must be emphasized, however, that this observation is from a single herd, and that breed (Jersey), certification status (organic), and milking system (AMS BouMatic) are fully confounded with farm in this design (Section 2.2). The 50% effect size at Farm B therefore cannot be statistically attributed to breed, organic management, or any other single factor in isolation; it should be regarded as a single-site observation that motivates a dedicated, adequately powered trial in certified organic herds before any generalization. Several non-exclusive explanations are consistent with the observed magnitude: higher baseline infection pressure typical of organic herds, where antibiotic metaphylaxis is unavailable; breed-specific differences in neutrophil function or vaginal microbiome ecology that have been reported between Jersey and Holstein cattle [54]; and a more favorable response to the specific LAB strains used. Comparable intravaginal probiotic interventions in commercial dairy herds have produced effect sizes in the same general range [30,55], suggesting that the magnitude observed here is plausible but not necessarily generalizable. The growing organic dairy sector in Canada, representing approximately 238 farms and over 1.58 million hectoliters of annual production during 2024/25 [56], lacks certified alternatives to antibiotics for uterine disease management, making the Farm B finding of immediate practical interest while underscoring the need for confirmation.

The absence of a statistically significant treatment effect on uterine involution, as assessed by transrectal palpation and ultrasonography at +2 and +4 weeks postpartum, is consistent with the established understanding that anatomical uterine regression, driven primarily by oxytocin-induced myometrial contractions, prostaglandin F2α pulses, and lochia expulsion, proceeds on a hormonally determined timeline that is largely independent of bacterial colonization status unless overt clinical metritis delays involution beyond the first 3 weeks [54,57]. The ROC analysis (Figure S2) confirmed excellent discriminatory accuracy for both gravid (AUC = 1.00) and non-gravid horns (AUC ≈ 0.98) by rectal palpation at the evaluated thresholds (*p* = 0.001), validating the measurement methodology. The positive association between PVD scores and uterine infection status (Figure 5; *p* < 0.001) confirms that purulent vaginal discharge serves as a reliable field biomarker for endometritis, in agreement with Sheldon et al. [3], and underscores the clinical utility of Metricheck scoring as a practical, non-invasive surveillance tool in commercial herd management.

The improvement in daily milk production in multiparous TRT3 cows (+4.6 L/day vs. TRT1 and +3.22 L/day vs. TRT2; *p* < 0.01 for both contrasts) is reported here as a mean difference rather than an odds ratio, as is appropriate for a continuous outcome. This effect is biologically plausible through at least three converging candidate mechanisms [58,59,60], although direct mechanistic verification was not part of the present trial. First, at the neuroendocrine level, circulating LPS has been shown to suppress hypothalamic growth hormone-releasing hormone (GHRH) pulsatility and to reduce pituitary prolactin secretion via TNF-α-mediated inhibition of anterior pituitary lactotrophs [61]; decreased uterine endotoxin burden would be predicted to restore prolactin tone and support alveolar milk synthesis. Second, subclinical endometritis is associated with prolongation of negative energy balance through cytokine-driven insulin resistance in peripheral adipose tissue and hepatic lipid mobilization, pathways that compete with mammary glucose partitioning for dietary energy substrates [62,63,64,65,66]. Third, decreased uterine infection incidence in TRT3 cows may have translated directly into fewer days of impaired feed intake, lower production losses, and earlier return to peak lactation, as has been documented in recent meta-analytic syntheses of the impact of metritis on lactation performance [67]. The improvement in milk production was parity-dependent (TRT × parity interaction; *p* = 0.01), with multiparous cows driving the effect, which is consistent with a stronger probiotic colonization advantage in cows whose vaginal microbiome had been previously shaped by exposure to the administered strains through co-housing across prior lactation cycles. The absence of a treatment effect on milk composition (fat, protein, lactose, SCC, MUN; all *p* > 0.05) is expected when volume gains are achieved primarily through restoration of baseline lactation physiology rather than enhancement of mammary secretory cell differentiation and is consistent with the prior literature on probiotic supplementation in transition cows [28,29].

Several limitations of the current study design merit explicit acknowledgment. First, the design used three treatment arms, two of which (TRT1: saline alone; TRT2: saline + skim-milk carrier) functioned as controls, but farm, breed, milking system, and organic certification were fully confounded. Second, no direct microbiome sequencing, culture-based enumeration, qPCR, or colonization data were generated, so mechanistic explanations related to microbiota modulation remain inferential. Third, some secondary diseases were uncommon, particularly displaced abomasum, limiting statistical power and widening confidence intervals. Fourth, milk fever was diagnosed clinically, but the study did not include blood calcium or endocrine measurements needed to determine whether treatment affected calcium metabolism. These limitations do not weaken the main uterine-health finding, but they require cautious interpretation of secondary disease outcomes.

## 5. Conclusions

In this multi-farm trial, intravaginal infusion of LAB probiotics around parturition was associated with a lower frequency of metritis/uterine infection, with the strongest and most consistent evidence observed for TRT3 relative to the two vehicle-control groups. The protective effect was concordant across metritis, subclinical mastitis, and retained placenta, and the metritis signal was additionally supported by the adjusted repeated-measures model; reporting of ORs, 95% CIs, and exact *p*-values indicates a biologically meaningful uterine-health signal, although the non-significant individual comparisons warrant cautious interpretation. Effects on milk fever, lameness, displaced abomasum, uterine involution, and reproductive performance were weaker, inconsistent, or exploratory. Because no direct microbiome, colonization, qPCR, culture, calcium, or inflammatory-biomarker data were collected, the proposed mechanisms involving pathogen exclusion, microbiota modulation, systemic inflammation, and calcium metabolism should be considered hypothesis-generating. Overall, the findings support intravaginal LAB as a promising antibiotic-free strategy for improving uterine health in transition dairy cows across commercial dairy systems.

## Figures and Tables

**Table 1:** Overall effect of intravaginally infused probiotics on six periparturient diseases in postpartum dairy cows.

OR vs. Pooled Controls (95% CI); Exact *p*-Value ^4^	TRT3 (*n* = 170) ^3^	TRT2 (*n* = 169) ^2^	TRT1 (*n* = 169) ^1^	Variable (%) (Case/Total)
0.69 (0.44–1.09); *p* = 0.119	18.8 (32/170)	24.9 (42/169)	25.4 (43/169)	Uterine infections
0.75 (0.32–1.72); *p* = 0.549	4.7 (8/170)	4.7 (8/169)	7.7 (13/169)	Retained placenta
—; *p* = 0.554	0.0 (0/170)	0.6 (1/169)	0.6 (1/169)	Displaced abomasum
0.57 (0.27–1.24); *p* = 0.163	5.3 (9/170)	8.3 (14/169)	9.5 (16/169)	Subclinical mastitis
0.81 (0.33–1.99); *p* = 0.825	4.1 (7/170)	5.3 (9/169)	4.7 (8/169)	Milk fever
1.20 (0.52–2.81); *p* = 0.663	5.3 (9/170)	4.1 (7/169)	4.7 (8/169)	Lameness

^1^ TRT1: sterile 0.9% saline (control). ^2^ TRT2: sterile 0.9% saline + sterile skim milk (control). ^3^ TRT3: LAB probiotic cocktail (*Lactobacillus sakei* FUA3089, *Pediococcus acidilactici* FUA3138, *Pediococcus acidilactici* FUA3140; 10^8^–10^9^ cfu/dose). Values are cow-level incidences calculated as cases divided by treatment-specific evaluable cows. Odds ratios compare TRT3 with pooled vehicle controls (TRT1 + TRT2) and are reported with 95% confidence intervals; ^4^ exact *p*-values are two-sided Fisher exact *p*-values. OR < 1 favors TRT3. CI: confidence interval.

**Table 2:** Frequencies of uterine infections postpartum among treatments in primiparous and multiparous cows.

Treatment	Uterine Infections (%) (Case/Total)			No Uterine Infections (%) (Case/Total)		
	P ^2^	M ^3^	Total (%)	P ^2^	M ^3^	Total (%)
TRT1 ^1^	30.0 (15/50)	23.5 (28/119)	25.4 (43/169)	70.0 (35/50)	76.5 (91/119)	74.6 (126/169)
TRT2 ^2^	22.4 (11/49)	25.8 (31/120)	24.9 (42/169)	77.6 (38/49)	74.2 (89/120)	75.1 (127/169)
TRT3 ^3^	25.0 (10/40)	16.9 (22/130)	18.8 (32/170)	75.0 (30/40)	83.1 (108/130)	81.2 (138/170)
Total	30.8 (36/117)	69.2 (81/117)	100.0	26.3 (103/391)	73.7 (288/391)	100.0

^1^ TRT1: sterile 0.9% saline. ^2^ TRT2: sterile 0.9% saline + sterile skim milk. ^3^ TRT3: LAB probiotic cocktail (10^8^–10^9^ cfu/dose). P: primiparous; M: multiparous.

**Table 3:** Frequencies of subclinical mastitis (>200 × 10^3^ cells/mL) postpartum among treatments in primiparous and multiparous cows.

Treatment	Subclinical Mastitis (%) (Case/Total)			No Subclinical Mastitis (%) (Case/Total)		
	P ^2^	M ^3^	Total (%)	P ^2^	M ^3^	Total (%)
TRT1 ^1^	4.0 (2/50)	11.8 (14/119)	9.5 (16/169)	96.0 (48/50)	88.2 (105/119)	90.5 (153/169)
TRT2 ^2^	8.2 (4/49)	8.3 (10/120)	8.3 (14/169)	91.8 (45/49)	91.7 (110/120)	91.7 (155/169)
TRT3 ^3^	5.0 (2/40)	5.4 (7/130)	5.3 (9/170)	95.0 (38/40)	94.6 (123/130)	94.7 (161/170)
Total	20.5 (8/39)	79.5 (31/39)	100.0	27.9 (131/469)	72.1 (338/469)	100.0

^1^ TRT1: sterile 0.9% saline. ^2^ TRT2: sterile 0.9% saline + sterile skim milk. ^3^ TRT3: LAB probiotic cocktail (10^8^–10^9^ cfu/dose). P: primiparous; M: multiparous.

**Table 4:** Frequencies of milk fever postpartum among treatments in primiparous and multiparous cows.

Treatment	Milk Fever (%) (Case/Total)			No Milk Fever (%) (Case/Total)		
	P ^2^	M ^3^	Total (%)	P ^2^	M ^3^	Total (%)
TRT1 ^1^	0.0 (0/50)	6.7 (8/119)	4.7 (8/169)	100.0 (50/50)	93.3 (111/119)	95.3 (161/169)
TRT2 ^2^	0.0 (0/49)	7.5 (9/120)	5.3 (9/169)	100.0 (49/49)	92.5 (111/120)	94.7 (160/169)
TRT3 ^3^	0.0 (0/40)	5.4 (7/130)	4.1 (7/170)	100.0 (40/40)	94.6 (123/130)	95.9 (163/170)
Total	0.0 (0/24)	100.0 (24/24)	100.0	28.7 (139/484)	71.3 (345/484)	100.0

^1^ TRT1: sterile 0.9% saline. ^2^ TRT2: sterile 0.9% saline + sterile skim milk. ^3^ TRT3: LAB probiotic cocktail (10^8^–10^9^ cfu/dose). P: primiparous; M: multiparous.

**Table 5:** Odds-ratio (OR) analyses for the incidence of uterine infections among treatments (TRT3 vs. TRT1 and TRT2) in primiparous and multiparous cows across the four farms.

Variable	TRT1 ^2^ vs. TRT3 ^4^			TRT2 ^3^ vs. TRT3 ^4^		
	OR ^1^	95% CL	*p*-Value	OR ^1^	95% CL	*p*-Value
UI primiparous	1.29	0.50–3.28	0.643	0.87	0.33–2.32	0.806
UI multiparous	1.51	0.81–2.82	0.208	1.71	0.93–3.16	0.091

^1^ OR = odds ratio; CL = confidence limits. ^2^ TRT1: sterile 0.9% saline. ^3^ TRT2: sterile 0.9% saline + sterile skim milk. ^4^ TRT3: LAB probiotic cocktail (10^8^–10^9^ cfu/dose). UI: uterine infection.

**Table 6:** Frequencies of retained placenta postpartum among treatments in primiparous and multiparous cows.

Treatment	Retained Placenta (%) (Case/Total)			No Retained Placenta (%) (Case/Total)		
	P ^2^	M ^3^	Total (%)	P ^2^	M ^3^	Total (%)
TRT1 ^1^	10.0 (5/50)	6.7 (8/119)	7.7 (13/169)	90.0 (45/50)	93.3 (111/119)	92.3 (156/169)
TRT2 ^2^	6.1 (3/49)	4.2 (5/120)	4.7 (8/169)	93.9 (46/49)	95.8 (115/120)	95.3 (161/169)
TRT3 ^3^	2.5 (1/40)	5.4 (7/130)	4.7 (8/170)	97.5 (39/40)	94.6 (123/130)	95.3 (162/170)
Total	31.0 (9/29)	69.0 (20/29)	100.0	27.1 (130/479)	72.9 (349/479)	100.0

^1^ TRT1: sterile 0.9% saline. ^2^ TRT2: sterile 0.9% saline + sterile skim milk. ^3^ TRT3: LAB probiotic cocktail (10^8^–10^9^ cfu/dose). P: primiparous; M: multiparous.

**Table 7:** Effect of intravaginal LAB probiotic cocktail on the incidence of periparturient diseases in postpartum Holstein dairy cows on Farm A.

Variable (%) (Case/Total)	TRT1 (*n* = 51) ^1^	TRT2 (*n* = 49) ^2^	TRT3 (*n* = 51) ^3^	Exact *p*-Value ^4^
Metritis	29.4 (15/51)	22.4 (11/49)	23.5 (12/51)	0.844
Subclinical mastitis	11.8 (6/51)	10.2 (5/49)	5.9 (3/51)	0.385
Milk fever	0	1	0	NS
Retained placenta	21.6 (11/51)	12.2 (6/49)	11.8 (6/51)	0.478
Displaced abomasum	0	1	0	NS
Lameness	7.8 (4/51)	8.2 (4/49)	5.9 (3/51)	0.751

^1^ TRT1: sterile 0.9% saline (control). ^2^ TRT2: sterile 0.9% saline + sterile skim milk (control). ^3^ TRT3: LAB probiotic cocktail (*Lactobacillus sakei* FUA3089, *Pediococcus acidilactici* FUA3138, *Pediococcus acidilactici* FUA3140; 10^8^–10^9^ cfu/dose). ^4^ The exact *p*-value is for the comparison of TRT3 versus pooled vehicle controls (two-sided Fisher’s exact test). NS: not significant; outcomes with very few cases are shown as case counts.

**Table 8:** Effect of intravaginal LAB probiotic cocktail on the incidence of periparturient diseases in postpartum organic Jersey dairy cows on Farm B.

Variable (%) (Case/Total)	TRT1 (*n* = 34) ^1^	TRT2 (*n* = 34) ^2^	TRT3 (*n* = 34) ^3^	Exact *p*-Value ^4^
Metritis	17.6 (6/34)	17.6 (6/34)	8.8 (3/34)	0.374
Subclinical mastitis	11.8 (4/34)	5.9 (2/34)	5.9 (2/34)	0.716
Milk fever	5.9 (2/34)	11.8 (4/34)	2.9 (1/34)	0.419
Retained placenta	0	0	0	NS
Displaced abomasum	0	0	0	NS
Lameness	2.9 (1/34)	2.9 (1/34)	5.9 (2/34)	0.599

^1^ TRT1: sterile 0.9% saline (control). ^2^ TRT2: sterile 0.9% saline + sterile skim milk (control). ^3^ TRT3: LAB probiotic cocktail (*Lactobacillus sakei* FUA3089, *Pediococcus acidilactici* FUA3138, *Pediococcus acidilactici* FUA3140; 10^8^–10^9^ cfu/dose). ^4^ The exact *p*-value is for the comparison of TRT3 versus pooled vehicle controls (two-sided Fisher’s exact test). NS: not significant; outcomes with very few cases are shown as case counts.

**Table 9:** Effect of intravaginal LAB probiotic cocktail on the incidence of periparturient diseases in postpartum Holstein dairy cows on Farm C.

Variable (%) (Case/Total)	TRT1 (*n* = 35) ^1^	TRT2 (*n* = 31) ^2^	TRT3 (*n* = 33) ^3^	Exact *p*-Value ^4^
Metritis	40.0 (14/35)	32.3 (10/31)	24.2 (8/33)	0.261
Subclinical mastitis	14.3 (5/35)	12.9 (4/31)	9.1 (3/33)	0.746
Milk fever	8.6 (3/35)	0.0 (0/31)	0.0 (0/33)	0.549
Retained placenta	1	1	0	NS
Displaced abomasum	0	0	0	NS
Lameness	5.7 (2/35)	6.5 (2/31)	9.1 (3/33)	0.683

^1^ TRT1: sterile 0.9% saline (control). ^2^ TRT2: sterile 0.9% saline + sterile skim milk (control). ^3^ TRT3: LAB probiotic cocktail (*Lactobacillus sakei* FUA3089, *Pediococcus acidilactici* FUA3138, *Pediococcus acidilactici* FUA3140; 10^8^–10^9^ cfu/dose). ^4^ The exact *p*-value is for the comparison of TRT3 versus pooled vehicle controls (two-sided Fisher’s exact test). NS: not significant; outcomes with very few cases are shown as case counts.

**Table 10:** Effect of intravaginal LAB probiotic cocktail on the incidence of periparturient diseases in postpartum Holstein dairy cows on Farm D.

Variable (%) (Case/Total)	TRT1 (*n* = 49) ^1^	TRT2 (*n* = 55) ^2^	TRT3 (*n* = 52) ^3^	Exact *p*-Value ^4^
Metritis	16.3 (8/49)	27.3 (15/55)	17.3 (9/52)	0.535
Subclinical mastitis	2.0 (1/49)	5.5 (3/55)	1.9 (1/52)	0.665
Milk fever	6.1 (3/49)	7.3 (4/55)	11.5 (6/52)	0.361
Retained placenta	2.0 (1/49)	1.8 (1/55)	3.8 (2/52)	0.601
Displaced abomasum	1	0	0	NS
Lameness	1	0	1	NS

^1^ TRT1: sterile 0.9% saline (control). ^2^ TRT2: sterile 0.9% saline + sterile skim milk (control). ^3^ TRT3: LAB probiotic cocktail (*Lactobacillus sakei* FUA3089, *Pediococcus acidilactici* FUA3138, *Pediococcus acidilactici* FUA3140; 10^8^–10^9^ cfu/dose). ^4^ The exact *p*-value is for the comparison of TRT3 versus pooled vehicle controls (two-sided Fisher’s exact test). NS: not significant; outcomes with very few cases are shown as case counts.

**Table 11:** Odds-ratio (OR) analyses for the effect of treatments (TRT3 vs. TRT1 and TRT2) on reproductive performance in dairy cows across the four farms.

Class	OR ^1^	95% CL	*p*-Value
TRT3 ^4^ vs. TRT1 ^2^	1.16	0.37–3.70	0.79
TRT3 ^4^ vs. TRT2 ^3^	1.61	0.54–4.81	0.38

^1^ OR = odds ratio; CL = confidence limits. ^2^ TRT1: Sterile 0.9% saline. ^3^ TRT2: Sterile 0.9% saline + sterile skim milk. ^4^ TRT3: LAB probiotic cocktail (10^8^–10^9^ cfu/dose).

**Table 12:** Milk composition of dairy cows from four farms treated with intravaginally infused lactic acid bacteria (LAB) around calving (median).

Variable	Treatments			Wks	TRT × Parity	
	TRT1 ^1^	TRT2 ^2^	TRT3 ^3^	*p*-Value ^a^	*p*-Value ^b^	*p*-Value ^c^
Fat (%)	3.73	4.25	3.72	0.35	0.75	0.71
Protein (%)	3.45	3.46	3.42	0.11	<0.01	0.22
Lactose (%)	4.33	4.35	4.38	0.92	<0.01	0.61
TS (%) ^4^	13.02	13.29	12.79	0.65	0.03	0.55
MUN (mg/dL) ^5^	10.65	10.25	10.40	0.37	0.01	0.37
SCC (10^3^/mL) ^6^	117	152	133	0.97	0.23	0.31

^1^ TRT1: sterile 0.9% saline. ^2^ TRT2: sterile 0.9% saline + sterile skim milk. ^3^ TRT3: LAB probiotic cocktail (10^8^–10^9^ cfu/dose). ^4^ TS: total solids. ^5^ MUN: milk urea nitrogen. ^6^ SCC: somatic cell count. SCC reported as the median owing to its right-skewed distribution. ^a^ *p*-value for treatment comparison. ^b^ *p*-value for week effect. ^c^ *p*-value for TRT × parity interaction.

## Data Availability

The data presented in this study are available on reasonable request from the corresponding author. The data are not publicly available due to confidentiality agreements with the participating dairy farms.

## Supplementary Materials

The following supporting information can be downloaded at: https://www.mdpi.com/article/10.3390/vetsci13060595/s1, Figure S1. REFLECT participant-flow diagram for the multi-farm intravaginal LAB probiotic trial; Figure S2. Receiver-operating characteristic (ROC) curves for rectal palpation as a diagnostic method for uterinehorn diameter assessment in postpartum dairy cows.
