# Supplementary material for: Intravaginal Probiotics in Transition Dairy Cows: A Randomized Multi-Farm Field Trial on Health and Milk Production
Source: Vet Sci. 2026 Jun 18;13(6):595. doi: 10.3390/vetsci13060595 (PMC13307743; doi:10.3390/vetsci13060595)
Supplement: Supplementary file 1 [file vetsci-13-00595-s001.zip › vetsci-4327078-supplementary.pdf]

Supplementary Materials

Intravaginal Probiotics in Transition Dairy Cows: A Randomized Multi-Farm Field Trial on Health and Milk Production

Rosales Barahona, E.; Garcia Dias, A. L.; Egyedy, A.; Ametaj, B. N.

Figure S1

Figure S2. REFLECT flow diagram: enrollment, randomization, allocation, follow-up, and analysis

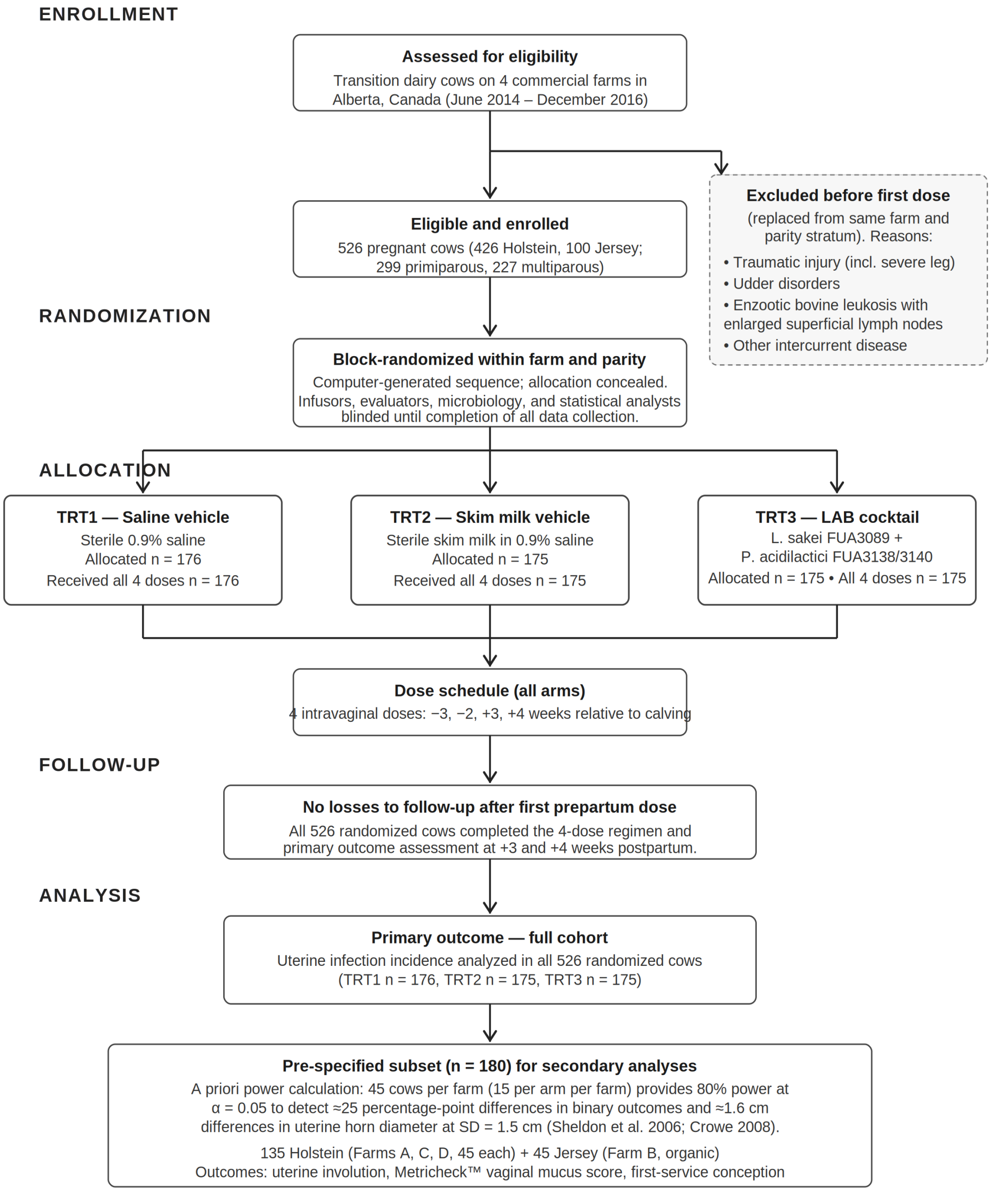

Reporting follows REFLECT (Sargeant et al., 2010) guidelines for randomized controlled trials in livestock and food safety.
Farms A, C, D: Holstein conventional (Farm A automatic milking system [AMS] DeLaval; Farm C AMS Lely; Farm D parlor 3× daily).
Farm B: Jersey, certified organic, AMS BouMatic.

Figure S1. REFLECT participant-flow diagram for the multi-farm intravaginal LAB probiotic trial, following the Reporting Guidelines for Randomized Controlled Trials in Livestock and Food Safety (REFLECT statement; Sargeant et al., 2010 [reference 32 in the main manuscript]). The diagram documents enrolment, allocation, follow-up, and analysis stages for n = 526 pregnant dairy cows (426 Holstein and 100 Jersey) recruited from four commercial Alberta farms (Farm A, Holstein, automatic milking system; Farm B, certified-organic Jersey, automatic milking system; Farm C, Holstein, automatic milking system; Farm D, Holstein, conventional parlor, 3×/day). Cows were block-randomized within farm and parity to TRT1 (sterile 0.9% saline + sterile skim milk; n = 176), TRT2 (sterile 0.9% saline alone; n = 175), or TRT3 (LAB probiotic cocktail — *Lactobacillus sakei* FUA3089, *Pediococcus acidilactici* FUA3138, *P. acidilactici* FUA3140; 10^8–10^9 cfu/dose, reconstituted in the same saline + skim-milk carrier as TRT1; n = 175). Each cow received four intravaginal doses of her assigned treatment at −3 and −2 weeks prepartum and at +3 and +4 weeks postpartum. No cows were lost to follow-up after randomization; replacement cows were enrolled in advance of calving to maintain the prespecified per-farm sample size and were assigned to their treatment arms by the same block-randomization procedure. Outcome ascertainment, sample collection, and laboratory analysis were performed blinded to treatment allocation, which was held by the project supervisor and not disclosed to on-farm staff, infusers, or evaluators until the analytical database was locked.

Figure S2

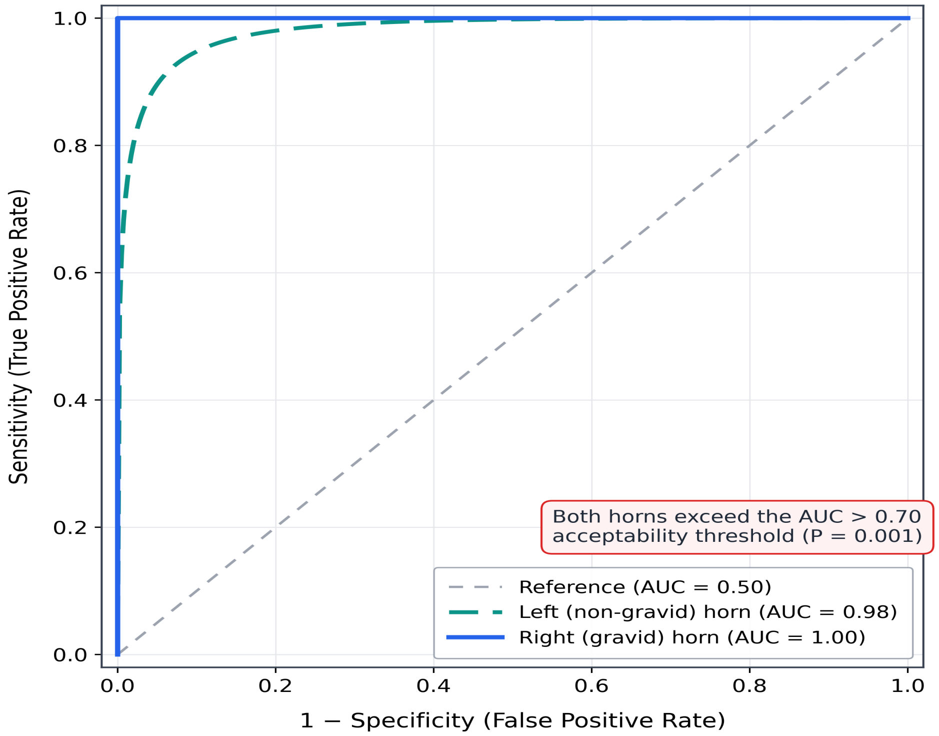

Figure S2. Receiver-operating characteristic (ROC) curves for rectal palpation as a diagnostic method for uterine horn diameter assessment in postpartum dairy cows. Solid blue line: right (gravid) uterine horn (AUC = 1.00); dashed teal line: left (non-gravid) uterine horn (AUC ≈ 0.98); grey dashed diagonal: chance reference line (AUC = 0.50). P = 0.001 for both horns. Both horns exceed the AUC > 0.70 acceptability threshold defined by Swets (1988) [reference 42 in the main manuscript], confirming that rectal palpation is an accurate, clinically acceptable method

for monitoring uterine involution in postpartum dairy cows. Diagnostic performance was assessed in *n* = 135 multiparous Holstein cows (Farms A, C, and D; 45 cows per farm) and *n* = 45 multiparous Jersey cows (Farm B) at +2 and +4 weeks postpartum, using a SonoSite® MicroMaxx ultrasound system (SonoSite Inc., Bothell, WA, USA) fitted with a 7.5 MHz probe as the reference standard against which palpation findings were compared. This figure originally appeared as Figure 4 in the submitted manuscript and has been moved to Supplementary Materials per Reviewer 2's recommendation (Comment 2.13), since the ROC validation of palpation methodology is methodological infrastructure rather than a core finding of the LAB intervention trial.
